# Supplementary figures and images for: Gene expression during THP-1 differentiation is influenced by vitamin D3 and not vibrational mechanostimulation
Source: PeerJ. 2021 Jul 14;9:e11773. doi: 10.7717/peerj.11773 (PMC8286059; doi:10.7717/peerj.11773)

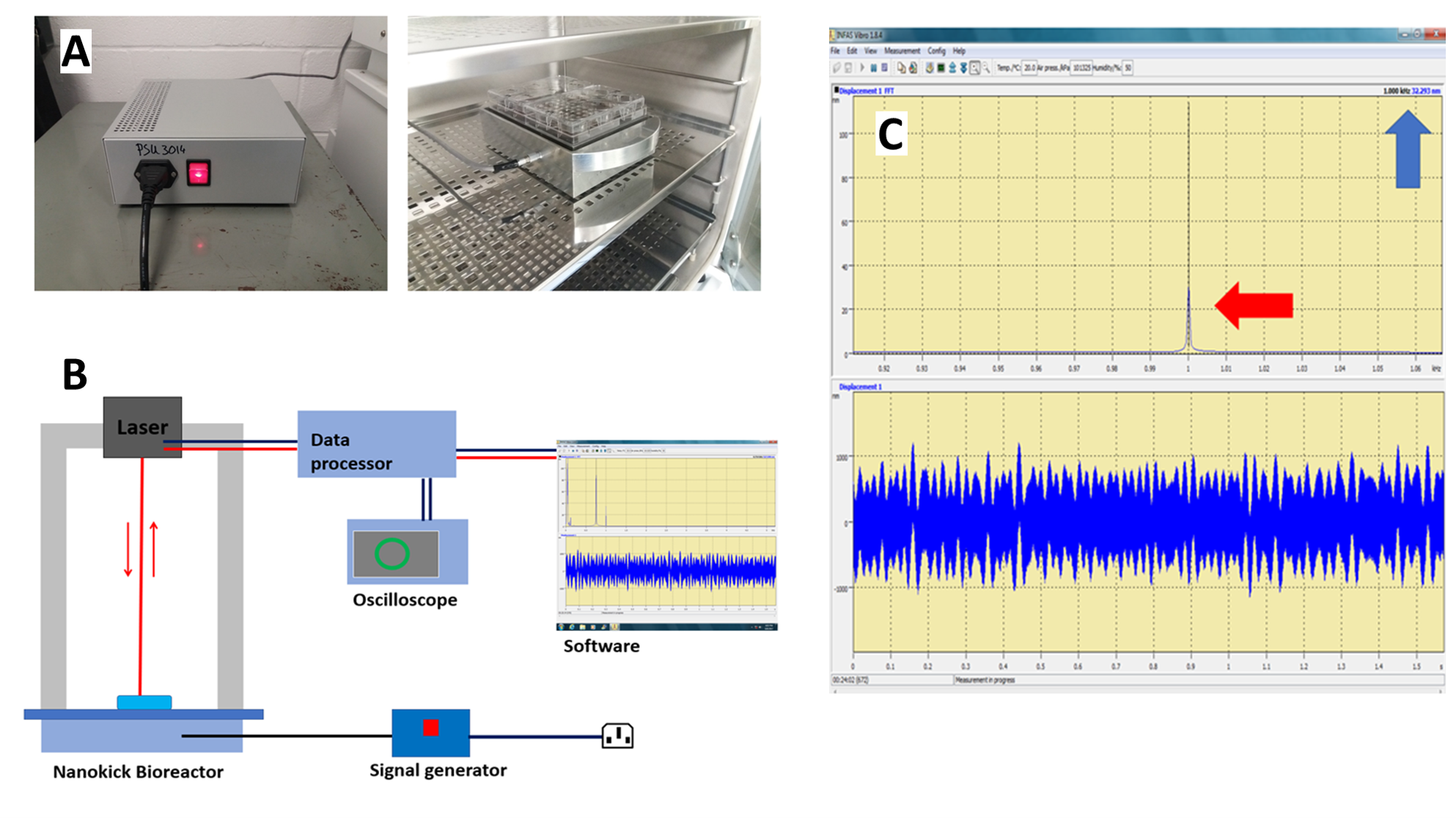

Supplement: Supplemental Information 1 — (A) Signal generator (left) and the Nanokick bioreactor in the incubator (right). Magnet-clamped plates can be seen on top of the bioreactor’s platform. (B) Laser interferometry was used to measure the frequency and the amplitude of the vibrations on the surface of the wells, which is the site where the mechanical stimulation was applied on the cells. Frequency and the amplitude of the vibrations were measured continuously over periods of 2–3 months, before and after experiments, to allow continuous assessment of the bioreactor’s functionality. The measurements were performed on the bioreactor twice; once after being left at room temperature (25 ° C) and another time after being incubated at 37 ° C for 24 h. During the laser interferometry, a continuous helium-neon beam (wavelength 632.8 nm) is reflected from the surface of the well at a distance of 25–30 cm and directed into the interferometer (SIOS Meßtechnik GmbH SP S-120) to create an interference pattern with a reference beam. Alignment of the beam is achieved by utilising an oscillator signal that appears as a circle on the oscilloscope’s screen. (C) The interferometers output is then analysed by INFAS Vibro computer software (SIOS Meßtechnik GmbH: Interferometry Analysis Software for Vibrometers), which performs a fast fourier transform (FFT) and plot the amplitude of motion in frequency space. The red arrow shows the frequency reading (1 kHz), and the blue arrow shows the amplitude of vibration (32.3 nm). [file peerj-09-11773-s001.png]

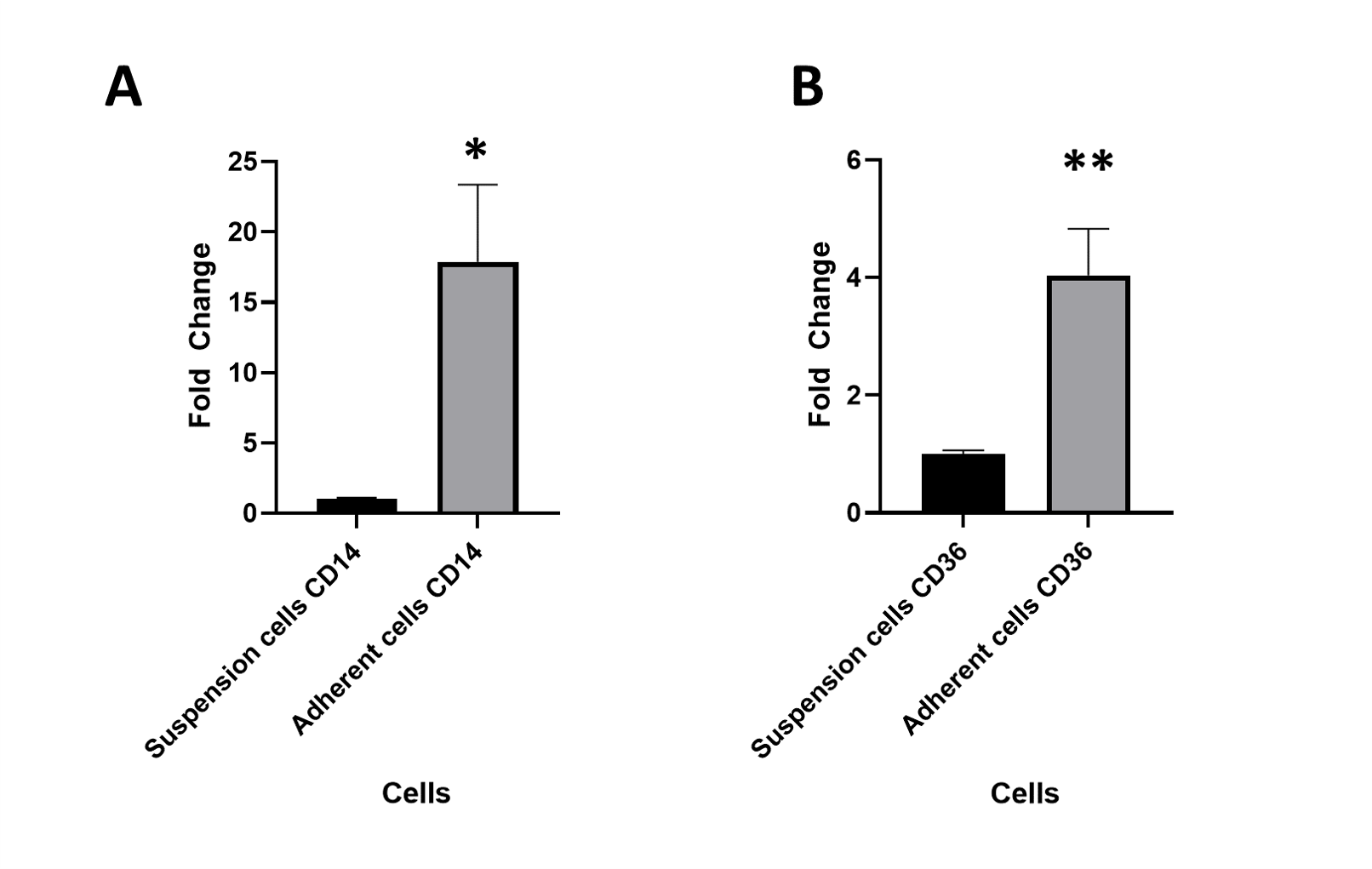

Supplement: Supplemental Information 2 — (A) Expression of CD14 mRNA in unstimulated adherent THP-1 cells ( N = 3) was 17.9 fold higher compared to the unstimulated suspension THP-1 cells ( N = 4) ( p value = 0.014). B) Expression of CD36 mRNA in unstimulated adherent THP-1 cells ( N = 3) was 4 fold higher compared to the unstimulated suspension THP-1 cells ( N = 4) ( p value = 0.006). Statistical analysis was performed using unpaired T test. Fold change was calculated using the ΔΔ Ct method. Fold change values higher than 1 indicate upregulation, whereas values between 0 and 1 indicate downregulation of mRNA transcripts. These results showed that the unstimulated THP-1 cells that had become adherent at 72 h, expressed higher macrophage markers than the unstimulated cells in suspension. This observation indicated differences between the suspension and adherent cells within the same population. Therefore, in this study the expression of genes upon stimulation was assessed in suspension and adherent cells separately, by comparing to the unstimulated suspension and adherent controls, respectively. [file peerj-09-11773-s002.png]

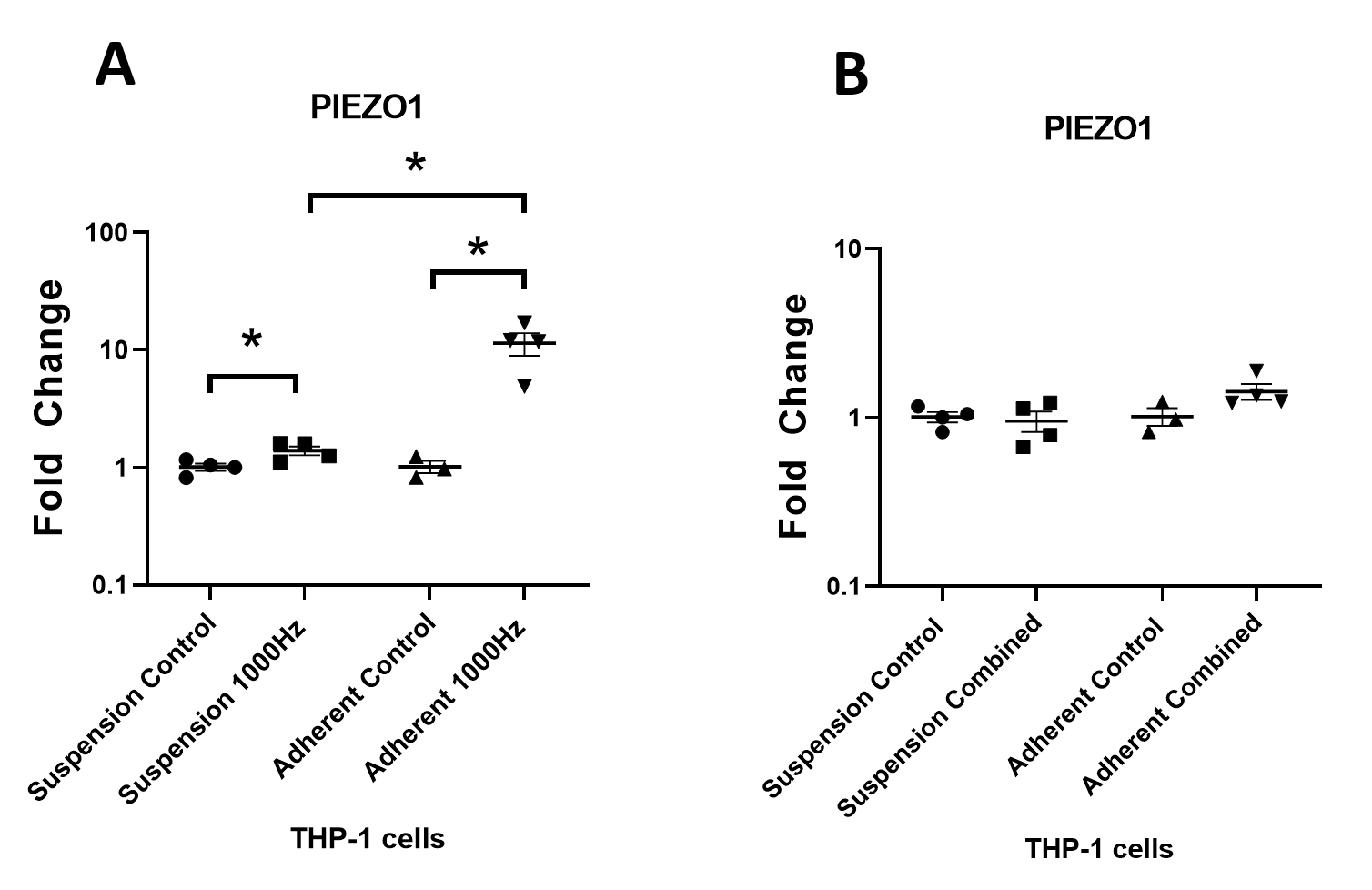

Supplement: Supplemental Information 3 — (A) PIEZO1 mRNA regulation in response to the 1,000 Hz vibrations. The 1,000 Hz vibrational stimulation upregulated PIEZO1 mRNA in stimulated suspension cells compared to the unstimulated suspension controls ( p∗ = 0.044), and in stimulated adherent cells compared to the unstimulated adherent controls ( p∗ = 0.025). The mean fold change value of PIEZO1 in the stimulated adherent cells was 8.2 fold higher than in the stimulated suspension cells ( p∗ = 0.027). This observation showed that the adherent cells, which were in contact with the vibrating surface of the well, responded by stronger upregulation of PIEZO1 mRNA compared to the floating cells. This could indicate potential involvement of mechanotransduction for the regulation of PIEZO1 expression in vibrated THP-1 monocytes. (B) PIEZO1 mRNA regulation in response to the combined stimulation with 50 nM of 1,25-dihydroxyvitamin D3 and 1,000 Hz vibrations. PIEZO1 mRNA in the stimulated suspension and adherent cells, were comparable to the unstimulated respective controls. No difference was recorded when comparing the fold change values between the stimulated suspension and stimulated adherent cells ( p = 0.061). Even though the application of the 1,000 Hz vibrations in isolation, resulted in strong upregulation of PIEZO1 in adherent cells (A), the vitamin D3 cancelled such effect in the combined treatment (B). The data presented as mean of four replicates ±SEM, with exception of adherent controls ( N = 3). The statistical analysis was performed using unpaired T test with Welch’s correction. P values lower than 0.05 were considered statistically significant. [file peerj-09-11773-s003.png]
